# Provider views on rapid diagnostic tests and antibiotic prescribing for respiratory tract infections: A mixed methods study

**DOI:** 10.1371/journal.pone.0260598

**Published:** 2021-11-29

**Authors:** Shana A. B. Burrowes, Tamar F. Barlam, Alexandra Skinner, Rebecca Berger, Pengsheng Ni, Mari-Lynn Drainoni

**Affiliations:** 1 Section of Infectious Diseases, Department of Medicine, Boston University School of Medicine, Boston, MA, United States of America; 2 Department of Health Law Policy and Management, Boston University School of Public Health, Boston, MA, United States of America; 3 Massachusetts Department of Public Health, Boston, MA, United States of America; 4 Biostatistics and Epidemiology Data Analytics Center (BEDAC) Boston University School of Public Health, Boston, MA, United States of America; 5 Evans Center for Implementation and Improvement Sciences (CIIS), Boston University School of Medicine, Boston, MA, United States of America; National and Kapodistrian University of Athens, GREECE

## Abstract

**Background:**

Respiratory tract infections (RTIs) are often inappropriately treated with antibiotics. Rapid diagnostic tests (RDTs) have been developed with the aim of improving antibiotic prescribing but uptake remains low. The aim of this study was to examine provider knowledge, attitudes and behaviors regarding RDT use and their relationship to antibiotic prescribing decisions across multiple clinical departments in an urban safety-net hospital.

**Methods:**

We conducted a mixed methods sequential explanatory study. Providers with prescribing authority (attending physicians, nurse practitioners and physician assistants) who had at least 20 RTI encounters from January 1, 2016 to December 31, 2018. Eighty-five providers completed surveys and 16 participated in interviews. We conducted electronic surveys via RedCap from April to July 2019, followed by semi-structured individual interviews from October to December 2019, to ascertain knowledge, attitudes and behaviors related to RDT use and antibiotic prescribing.

**Results:**

Survey findings indicated that providers felt knowledgeable about antibiotic prescribing guidelines. They reported high familiarity with the rapid streptococcus and rapid influenza tests. Familiarity with comprehensive respiratory panel PCR (RPP-respiratory panel PCR) and procalcitonin differed by clinical department. Qualitative interviews identified four main themes: providers trust their clinical judgment more than rapid test results; patient-provider relationships play an important role in prescribing decisions; there is patient demand for antibiotics and providers employ different strategies to address the demand and providers do not believe RDTs are implemented with sufficient education or evidence for clinical practice.

**Conclusion:**

Prescribers are knowledgeable about prescribing guidelines but often rely on clinical judgement to make final decisions. The utility of RDTs is specific to the type of RDT and the clinical department. Given the low familiarity and clinical utility of RPP and procalcitonin, providers may require additional education and these tests may need to be implemented differently based on clinical department.

## Introduction

Respiratory tract infections (RTIs) account for approximately 60% of antibiotic prescriptions in the outpatient setting [1,2] although the majority of RTIs are viral [2–4], and antibiotic treatment is not indicated [3,5,6]. One-third to one-half of acute RTIs in adults are inappropriately treated with antibiotics [7–10] with acute bronchitis and acute sinusitis accounting for a large proportion of those [6,11,12]. In pediatrics, more than 70% of antibiotics are prescribed in the outpatient setting and approximately 30% are inappropriately prescribed [13]. Pediatric patients with viral infections such as bronchitis and bronchiolitis are often given antibiotics, even in the absence of bacterial infection [14,15]. Inappropriate prescribing promotes antibiotic resistance and increases healthcare costs [2,16]. In addition, antibiotics are frequently associated with side effects for patients [16,17].

Inappropriate prescription of antibiotics is a multifaceted problem, and providers report that both clinical and non-clinical factors contribute to their prescribing decisions [18,19]. In the US, Patel et al. found that clinical factors such as duration and severity of symptoms heavily influenced prescribing decisions, particularly among high-prescribing providers [20]. In primary care clinics in Boston, clinicians stated that diagnostic uncertainty drives prescribing despite awareness of clinical guidelines [6]. Other studies indicate that providers prefer to rely on their clinical judgment over prescribing guidelines [18,19]. Non-clinical factors such as lack of motivation to change practice, time constraints, patient pressure and pressure from pharmaceutical companies also influence antibiotic prescribing [18–21].

One strategy to improve prescribing has been the development and implementation of rapid diagnostic tests (RDTs) for RTIs based on the belief that if providers have accurate diagnoses quickly, it will improve prescribing [5]. Prior research by our team has shown that RDT use is low and clinical utility and impact on prescribing is varied [22], consistent with other reports [23–25]. In another study, providers in U.S. family medicine clinics had mixed attitudes about RDTs, citing inaccuracy, over-reliance on tests and costs as barriers to use [26]. Similarly, internal medicine practitioners in a Dutch hospital cited inaccuracy and cost as barriers to use of RDTs but highlighted that patient factors and the need for clear clinical guidelines are also important in their decision to use tests [27].

Beyond these limited data, little is known about why providers do or do not use RDTs and how their use or lack of use influences prescribing decisions [5,18,19,26]. We sought to examine provider knowledge, attitudes and behaviors regarding RDT use and their relationship to antibiotic prescribing decisions across multiple clinical departments in an urban safety-net hospital.

## Methods

### Ethics statement

Patient data was not utilized in these analyses. All questionnaire and survey data were anonymized prior to analysis. The Boston University Medical Campus Institutional Review Board approved all study activities. All participants provided informed consent. Participants consented to the survey through the email invite. Participants opted in to the qualitative interview at the end of the survey and gave oral consent at the beginning of the interview.

### Design, setting and participants

We conducted a mixed methods sequential explanatory study [28]. First, we analyzed data from the electronic health record (EHR) for all outpatient, urgent care and emergency department (ED) visits with a primary diagnosis of an RTI from January 1, 2016 to December 31, 2018. Data included, but were not limited to, RDTs used, antibiotics prescribed, and patient and provider demographic information. We included the following four RDTs: the rapid streptococcal antigen test (OSOM Ultra Strep A) which qualitatively detects group A streptococcus (GAS); the comprehensive respiratory panel PCR (RPP-respiratory panel PCR) (FilmArray Respiratory Panel) that tests for 20 respiratory pathogens (17 viruses and 3 bacteria); a rapid influenza test; and a rapid test for procalcitonin (PCT) which measure PCT in serum or plasma. Further details on data extraction and the results of these analyses have been previously published [22]. In this paper we conducted a survey followed by individual interviews with providers to obtain additional context about their knowledge, attitudes and behaviors related to RDT use and antibiotic prescribing. Our survey included providers with prescribing authority (attending physicians, nurse practitioners (NPs) and physician assistants (PAs)) who had at least 20 RTI encounters over the three-year study period. Patient data was not utilized in these analyses. The Boston University Medical Campus Institutional Review Board approved all study activities.

### Survey development and data collection

The survey was based on the core constructs in the Cabana et al conceptual framework of provider barriers to following clinical practice guidelines [29]. Questions assessed providers’ knowledge, attitudes and behaviors related to antibiotic prescribing for RTIs and the use of RDTs. The survey was initially pilot tested with 10 providers outside of the study healthcare system and refined based on their feedback. The final instrument comprised 13 questions on antibiotic prescribing, and 8 questions on RDTs. Each RDT question was posed separately for the four RDTs of interest (i.e. Rapid GAS, Rapid Influenza, RPP and Procalcitonin) (See S1 Appendix). Responses were recorded on a six-point Likert scale as follows: Strongly Agree, Agree, Neither Agree or Disagree, Disagree, Strongly Disagree, I Don’t Know. Survey invitations were sent via REDCap and the survey was administered via REDCap between April and July 2019. Providers received a maximum of four reminders to complete the survey. The first three reminders were sent at two-week intervals. The final reminder was sent one month after the third reminder. At the completion of the survey, providers were asked if they would be willing to opt in to participate in a follow-up qualitative interview.

### Qualitative interview development and data collection

The semi-structured qualitative interview guide was based on the Cabana framework and findings from the survey. The interview guide was piloted with four providers to determine length of the interview and acceptability. The final interview guide comprised five questions, each with several probes for discussion (See S2 Appendix). Questions about prescribing were aimed at understanding providers’ processes, attitudes and beliefs about prescribing antibiotics, including but not limited to patient-level clinical factors and external factors that may play a role. Questions about RDTs probed the provider’s personal experience with the RDTs available at our institution and their views on overall use. We also paid special attention to their views regarding clinical utility of rapid diagnostics in bronchitis and sinusitis cases, given the high proportion of antibiotic use for these diagnoses. All interviews were conducted by telephone and audio recorded with participants’ oral consent. All interviews were conducted by one team member, S.B, who is trained in qualitative interview techniques and knowledgeable about rapid diagnostic testing and antibiotic stewardship. Interviews occurred between October and December 2019 and averaged 25 minutes (range 19–31 minutes).

#### Data analysis

*Surveys*. Completed surveys were anonymized and exported from REDCap for analysis. Univariate analysis examined the distribution of responses for each question separately using Chi Square and Fisher Exact tests. Analysis showed small cell sizes across the six response categories; we therefore combined the responses of Strongly Agree and Agree into one category, and similarly combined responses of Strongly Disagree and Disagree. When analyzing the responses to questions on RDTs, each RDT was assessed separately. Bivariate analysis examined the distribution of responses by clinical department and provider type. Given small cell sizes, department type was reduced to the following categories: emergency department, pediatrics, and other (combination of internal medicine, family medicine and otolaryngology). All statistical analyses were conducted using SAS (version 9.4; SAS Institute Inc., Cary, NC, USA). Testing was two-sided and done at 0.05 level significance.

*Qualitative interviews*. Twenty-two providers agreed to participate in follow-up telephone interviews; 16 completed interviews. We conducted interviews until thematic saturation. The audio-recorded interviews were anonymized and transcribed by a professional transcription company and checked for accuracy by the interviewer. Transcripts were imported into NVivo, version 12 for coding and data analysis. We conducted a directed content analysis [30] using the constructs of the Cabana framework. Knowledge, attitudes and behaviors were our parent codes with sub-codes as outlined in the framework [29]. For example, knowledge was subcategorized using the child codes of awareness and familiarity, which were then further subcategorized into grandchild codes that can impact knowledge such as access to guidelines (See S1 Fig). The study team reviewed the preliminary codebook; S.B, A.S and R.B then each independently coded the same transcript. Coding was reviewed and compared line by line and any inconsistencies were resolved. All members of the team (S.B, A.S, R.B, T.F.B and M.D) then coded two more transcripts independently. The transcripts were reviewed, inconsistencies were addressed, and the final codes confirmed. The remaining transcripts were coded independently by S.B, A.S and R.B and reviewed. Any coding conflicts were resolved in consultation with M.D.

## Results

### Overview of the survey sample

Surveys were initially sent to 200 providers; 35 were no longer at the institution and eight were on leave, leaving an effective sample of 157. The response rate was 54% (N = 85). Survey non-responders were more likely to be female but did not differ by any other demographic factors. Table 1 provides information on the survey sample. Respondents were predominantly white (67%), female (60%), and physicians (86%). Respondents worked primarily in Pediatrics (34%), Internal Medicine (31%) and the ED (27%). Forty-six providers (54%) had worked in the hospital for five years or less and 45 (53%) had more than 100 visits in which an RTI was diagnosed during the study period.

**Table 1:** Demographic description of survey and interview participants.

Variable	Survey Participants/N = 85 (%)	Interview Participants/N = 16 (%)	p value^a^
**Sex**			
Male	33 (39)	8 (50)	0.65
Female	51 (60)	8 (50)	
Prefer not to answer	1 (1)	0	
**Age Group**			
< = 45	43 (51)	6 (38)	0.10
46–55	22 (26)	2 (12)	
56–65	14 (16)	4 (25)	
>65	6 (7)	4 (25)	
**Race**			
Non-Hispanic White	57 (67)	12 (75)	0.90
African American/Black	4 (5)	0	
Hispanic	3 (3)	0	
Asian	11 (13)	3 (19)	
Other	4 (5)	1 (6)	
Prefer not to answer	6 (7)	0	
**Degree**			
MD/DO	73 (86)	13 (81)	0.70
NP	12 (14)	3 (19)	
**Department**			
Internal Medicine	26 (31)	5 (31)	0.61
Emergency Department	23 (27)	3 (19)	
Pediatrics	29 (34)	5 (31)	
Other	7 (8)	3 (19)	
**Years employed at institution**			
< = 5	46 (54)	4 (25)	0.09
6–20	25 (29)	7 (44)	
>20	14 (17)	5 (31)	
**Number of patient encounters from 2016–2018**			
< = 100	40 (47)	8 (50)	0.45
101–500	37 (44)	5 (31)	
>500	8 (9)	3 (19)	

^**a**^ Chi Square or Fisher Exact p value.

### Survey findings

#### Antibiotic prescribing

Over 90% of providers indicated that they were adequately trained to prescribe antibiotics and were familiar with clinical guidelines related to antibiotic treatment of acute RTIs. When posed knowledge questions to ascertain prescribing decisions based on clinical presentation and rapid diagnostic test results, 81–85% of providers gave answers that were consistent with clinical guidelines (Table 2). Though providers were not sure how their prescribing compared to others in their department, they believed that antibiotic prescribing consistency across their department was important. When making their prescribing decisions, they considered the well-being of the patient to be very important, with 79% stating that not missing a possible bacterial infection was a high priority and 82% noting that avoiding adverse effects of antibiotics was an important factor in their prescribing decisions. Thirty-six (42%) providers thought that patient demand for antibiotics was a significant issue in their practice, although this differed by department. Time constraints to educate was viewed as an issue in 42% of all providers across all provider types with 31% of pediatric providers, 48% of ED and other providers viewing this as a barrier” (See S1 Table). Providers did agree, regardless of department, that patients are not well informed about antibiotics and that patients believe antibiotics are necessary for most RTIs (Table 2).

**Table 2:** Survey responses assessing knowledge and attitudes about antibiotic prescribing for respiratory tract infections.

Antibiotic Prescribing (n = 85)	Disagree or Strongly disagree n (%)	Agree or Strongly Agree n (%)	Neither agree or disagree n (%)	I don’t Know n (%)
I am adequately trained to prescribe antibiotics	1 (1.2)	82 (96.5)	2 (2.3)	
I prescribe antibiotics more appropriately than other providers in my department or section. ^a^	6 (7.2)	18 (21.4)	42 (50)	18 (21.4)
It is important to me that my prescribing is consistent with others in my department or section. ^a^	13 (15.5)	49 (58.3)	22 (26.2)	
I often overprescribe antibiotics.	67 (78.8)	4 (4.7)	14 (16.5)	
It is a high priority for me not to miss a possible bacterial infection when I select antibiotics to treat my patients.	6 (7.1)	67 (78.8)	12 (14.1)	
Avoiding adverse effects of antibiotics, such as Clostridium difficile infection, is a major consideration when I prescribe antibiotics.	5 (5.9)	70 (82.3)	10 (11.8)	
Patient demand for antibiotics is a significant issue in my practice.	31 (36.5)	36 (42.3)	18 (21.2)	
Patients are well informed about appropriate antibiotic use.	59 (69.4)	8 (9.4)	18 (21.2)	
Patients believe antibiotics are necessary for most respiratory tract infections.	20 (23.5)	50 (58.8)	15 (17.7)	
I have sufficient time to educate patients when antibiotics are not needed for their infection.	36 (42.4)	33 (38.8)	16 (18.8)	
I am familiar with clinical guidelines related to antibiotic treatment for acute respiratory tract infections.	3 (3.5)	77 (90.6)	5 (5.9)	
Patients with acute bronchitis should get antibiotics if their sputum becomes yellow or green.	69 (81.1)	2 (2.4)	12 (14.1)	2 (2.4)
Antibiotics should not be prescribed for clinically stable patients with a comprehensive respiratory panel positive for RSV.	4 (4.7)	72 (84.7)	6 (7.1)	3 (3.5)

^**a**^Questions with missing data from respondents.

#### Rapid diagnostic tests for RTIs

Familiarity with and views on rapid diagnostic testing procedures varied by RDT type. Over 90% of providers, regardless of department or training, were familiar with the rapid GAS and rapid influenza tests. They trusted the test results from these RDTs, thought that results were available in a timely manner and helped them to prescribe better (Table 3). Unlike the rapid GAS and rapid influenza tests, familiarity with RPP and procalcitonin differed by department (Table 4). In the ED, familiarity with RPP (100%) and procalcitonin (91%) was high. Although ED providers thought that test results were available quickly enough for both RDTs, they found only RPP and not PCT was helpful in making antibiotic prescribing decisions. Pediatric providers were also familiar with RPP (100%) but less so for procalcitonin (61%). Only RPP and not PCT results were available quickly and helped guide prescribing. In other departments, familiarity with procalcitonin was limited (39%), and providers either did not find the test clinically useful or had no views on its utility (Table 4). For all RDTs there was no agreement on whether test results should supersede clinical judgement (Table 3).

**Table 3:** Survey responses assessing knowledge and attitudes about rapid diagnostic tests for respiratory tract infections.

**Rapid Diagnostic Tests (n = 85)**	**Disagree or Strongly disagree n (%)**	**Agree or Strongly Agree n (%)**	**Neither agree or disagree n (%)**	**I don’t Know n (%)**	
**I am familiar with rapid diagnostic testing procedures available at Boston Medical Center.** ^a^					
Respiratory Panel PCR	9 (10.7)	74 (88.1)	1 (1.2)		
Rapid Streptococcal Testing	4 (4.8)	78 (94)	1 (1.2)		
Rapid Influenza Testing	6 (7.2)	76 (91.6)	1 (1.2)		
Procalcitonin	24 (29.2)	50 (61)	8 (9.8)		
**Rapid diagnostic tests for infectious diseases help me make better antibiotic prescribing decisions.**					
Respiratory Panel PCR	6 (7.1)	63 (74.1)	16 (18.8)		
Rapid Streptococcal Testing	6 (7.1)	75 (88.2)	4 (4.7)		
Rapid Influenza Testing^a^	4 (4.8)	73 (88)	6 (7.2)		
Procalcitonin	13 (15.3)	29 (34.1)	43 (50.6)		
**Rapid diagnostic testing results are available quickly enough to help guide my antibiotic prescribing decisions.** ^**a**^					
Respiratory Panel PCR	11 (13.1)	49 (58.3)	24 (28.6)		
Rapid Streptococcal Testing	5 (6)	68 (81)	11 (13)		
Rapid Influenza Testing	10 (12.1)	62 (74.7)	11 (13.2)		
Procalcitonin	10 (12.3)	29 (35.8)	42 (51.9)		
**I seldom change antibiotic decisions based on rapid diagnostic testing.**					
Respiratory Panel PCR	29 (34.1)	31 (36.5)	25 (29.4)		
Rapid Streptococcal Testing	48 (56.5)	25 (29.4)	12 (14.1)		
Rapid Influenza Testing	42 (49.4)	27 (31.8)	16 (18.8)		
Procalcitonin	19 (22.4)	19 (22.4)	47 (55.2)		
**I do not trust the results of many of the rapid diagnostic tests.** ^a^					
Respiratory Panel PCR	70 (83.3)	3 (3.6)	11 (13.1)		
Rapid Streptococcal Testing	61 (72.6)	10 (11.9)	13 (15.5)		
Rapid Influenza Testing	67 (79.8)	4 (4.8)	13 (15.4)		
Procalcitonin	49 (59)	4 (4.9)	30 (36.1)		
	**Disagree or Strongly disagree n (%)**	**Agree or Strongly Agree n (%)**	**Neither agree or disagree n (%)**	**I don’t Know n (%)**	
**Rapid diagnostic testing results should never supersede my clinical assessment.** ^a^					
Respiratory Panel PCR	29 (34.5)	33 (39.3)	22 (26.2)		
Rapid Streptococcal Testing	30 (35.7)	28 (33.3)	26 (31)		
Rapid Influenza Testing	29 (35)	29 (35)	25 (30)		
Procalcitonin	16 (19)	32 (38.1)	36 (42.9)		
	**Cost**	**Specificity**	**Sensitivity**	**Time to results**	**Other**
**What is the most important factor you consider when deciding whether to order a rapid diagnostic test for your patient?** ^a^					
Respiratory Panel PCR	9 (11.2)	14 (17.5)	25 (31.2)	15 (18.9)	17 (21.2)
Rapid Streptococcal Testing		18 (22.2)	30 (37.1)	18 (22.2)	15 (18.5)
Rapid Influenza Testing	2 (2.4)	18 (22)	27 (32.9)	20 (24.4)	15 (18.3)
Procalcitonin	9 (11.6)	17 (21.8)	20 (25.6)	10 (12.8)	22 (28.2)
**What is the least important factor you consider when deciding whether to order a rapid diagnostic test for your patient?** ^a^					
Respiratory Panel PCR	37 (46.8)	4 (5.1)	8 (10.1)	19 (24.1)	11 (13.9)
Rapid Streptococcal Testing	44 (54.3)	7 (8.6)	4 (5)	15 (18.5)	11 (13.6)
Rapid Influenza Testing	42 (51.8)	6 (7.4)	5 (6.2)	17 (21)	11 (13.6)
Procalcitonin	36 (47.4)	5 (6.6)	5 (6.6)	16 (21)	14 (18.4)

^**a**^ Questions with missing data from respondents.

**Table 4:** Survey responses assessing knowledge and attitudes about rapid diagnostic tests for respiratory tract infections by department and provider type.

**I am familiar with rapid diagnostic testing procedures available at Boston Medical Center** ^a^			
	**Disagree or Strongly disagree n (%)**	**Agree or Strongly Agree n (%)**	**Neither agree or disagree n (%)**
**Department**	**Respiratory Panel PCR**		
Emergency Department	0	23 (100)	0
Pediatrics	0	29 (100)	0
Other	9 (28.1)	22 (68.8)	1 (3.1)
**Provider Type**			
MD	7 (9.7)	64 (88.9)	1 (1.4)
NP	2 (16.7)	10 (83.3)	0
**Department**	**Rapid Streptococcal Testing**		
Emergency Department	0	23 (100)	0
Pediatrics	1 (3.6)	27 (96.4)	0
Other	3 (9.4)	28 (87.5)	1 (3.1)
**Provider Type**			
MD	4 (5.6)	66 (93)	1 (1.4)
NP	0	12 (100)	0
**Department**	**Rapid Influenza Testing**		
Emergency Department	0	23 (100)	0
Pediatrics	1 (3.4)	28 (96.6)	0
Other	5 (16.1)	25 (80.7)	1 (3.2)
**Provider Type**			
MD	6 (8.5)	64 (90.1)	1 (1.4)
NP	0	12 (100)	0
**Department**	**Procalcitonin**		
Emergency Department	1 (4.4)	21 (91.3)	1 (4.4)
Pediatrics	7 (25)	17 (60.7)	4 (14.3)
Other	16 (51.6)	12 (38.7)	3 (9.7)
**Provider Type**			
MD	20 (28.6)	44 (62.9)	6 (8.6)
NP	4 (33.3)	6 (50)	2 (16.7)
**Rapid diagnostic tests for infectious diseases help me make better antibiotic prescribing decisions.**			
**Department**	**Respiratory Panel PCR**		
Emergency Department	2 (8.7)	20 (87)	1 (4.3)
Pediatrics	0	24 (82.8)	5 (17.2)
Other	4 (12.1)	19 (57.6)	10 (30.3)
**Provider Type**			
MD	6 (8.2)	51 (69.9)	16 (21.9)
NP	0	12 (100)	0
**Department**	**Rapid Streptococcal Testing**		
Emergency Department	3 (13)	20 (87)	0
Pediatrics	0	28 (96.6)	1 (3.4)
Other	3 (9.1)	27 (81.8)	3 (9.1)
**Provider Type**			
MD	6 (8.2)	63 (86.3)	4 (5.5)
NP	0	12 (100)	0
**Department**	**Rapid Influenza Testing** ^**a**^		
Emergency Department	3 (13.6)	19 (86.4)	0
Pediatrics	0	27 (93.1)	2 (6.9)
Other	1 (3.1)	27 (84.4)	4 (12.5)
**Provider Type**			
MD	4 (5.6)	61 (85.9)	6 (8.5)
NP	0	12 (100)	0
**Department**	**Procalcitonin**		
Emergency Department	6 (26.1)	9 (39.1)	8 (34.8)
Pediatrics	2 (6.9)	11 (37.9)	16 (55.2)
Other	5 (15.1)	9 (27.3)	19 (57.6)
**Provider Type**			
MD	13 (17.8)	24 (32.9)	36 (49.3)
NP	0	5 (41.7)	7 (58.3)
**Rapid diagnostic testing results are available quickly enough to help guide my antibiotic prescribing decisions.** ^**a**^			
**Department**	**Respiratory Panel PCR**		
Emergency Department	3 (13)	16 (69.6)	4 (17.4)
Pediatrics	4 (13.8)	21 (72.4)	4 (13.8)
Other	4 (12.5)	12 (37.5)	16 (50)
**Provider Type**			
MD	9 (12.5)	42 (58.3)	21 (29.2)
NP	2 (16.7)	7 (58.3)	3 (25)
**Department**	**Rapid Streptococcal Testing**		
Emergency Department	1 (4.3)	20 (87)	2 (8.7)
Pediatrics	0	27 (93.1)	2 (6.9)
Other	4 (12.5)	21 (65.6)	7 (21.9)
**Provider Type**			
MD	3 (4.2)	59 (81.9)	10 (13.9)
NP	2 (16.7)	9 (75)	1 (8.3)
**Department**	**Rapid Influenza Testing**		
Emergency Department	3 (13.6)	18 (81.8)	1 (4.6)
Pediatrics	3 (10.3)	23 (79.3)	3 (10.3)
Other	4 (12.5)	21 (65.6)	7 (21.9)
**Provider Type**			
MD	9 (12.7)	53 (74.6)	9 (12.7)
NP	1 (8.3)	9 (75)	2 (16.7)
**Department**	**Procalcitonin**		
Emergency Department	3 (13.6)	14 (63.6)	5 (22.7)
Pediatrics	2 (7.1)	8 (28.6)	18 (64.3)
Other	5 (16.1)	7 (22.6)	19 (61.3)
**Provider Type**			
MD	8 (11.6)	27 (39.1)	34 (49.3)
NP	2 (16.7)	2 (16.7)	8 (66.7)

^**a**^ Questions with missing data from respondents.

### Overview of the qualitative interview sample

Participants in the qualitative interviews were primarily MDs (81%), white (75%), less than 45 years of age and worked in Internal Medicine or Pediatrics. Unlike the survey participants, the majority of providers were employed at the institution for longer, at least 11 years (63%). Over half had more than 100 visits in which an RTI was diagnosed during the study period of interest.

### Qualitative interview themes

We identified four primary themes in provider interviews: 1) providers trust their clinical judgment more than rapid test results; 2) patient-provider relationships play an important role in prescribing decisions; 3) there is patient demand for antibiotics and providers employ different strategies to address the demand; and 4) providers do not believe RDTs are implemented with sufficient education or evidence for clinical practice. Each of these themes is described below with illustrative quotes.

#### 1) Providers trust their clinical judgment more than rapid test results

Providers use RDTs infrequently and, even when ordered, ultimately rely on their clinical impression. Test results seldom overrode clinical judgment when making prescribing decisions. “I’ll do a rapid screen to help tell me if it really is strep or not. But it’s not going to change what I do…there’s a lot of other bacteria that can cause these infections. And if I actually see pus, then I’m going to treat it. So I’ll treat that with an antibiotic.” Providers also describe how they find tests to be useful only when the clinician cannot make the decision based purely on clinical judgement (i.e. tiebreaker). “I think they are helpful in certain situations…I try not to use them unless my clinical judgment suggested I would be helped by the answer.”

#### 2) Patient-provider relationships play an important role in prescribing decisions

The relationship between the patient and the provider can influence antibiotic prescribing. For some providers they are more likely to prescribe an antibiotic if they know and trust their patient. “If you know the patient, sometimes I feel like you’re more inclined to give them an antibiotic. …You don’t know the patient? That’s more difficult. Because you don’t know what the activities are, what they’re going to do, anything…” This point was noted by other providers, “I guess the patients I know very well I might be willing to prescribe over the phone if it really seems like it sounds like a sinusitis or something…”

#### 3) There is patient demand for antibiotics and providers employ different strategies to address the demand

Patient demand for antibiotics can lead providers to prescribe when they otherwise would not. Some providers described giving alternative treatments in lieu of an antibiotic and this not only addresses demand but improves patient satisfaction. “… I think it can sometimes be where if you don’t prescribe antibiotics, the patient may feel like you’re not giving them something to help. You don’t want to help them… I say… Here is what you should expect and here are the things that will help you, like certain over the counter medications, maybe using the vaporizer sort of non-pharmacologic treatments.” Another provider also stated, “I’d just prescribe something that’s basically a placebo or mild…because people usually don’t want to walk away without anything…” On the other hand, some providers think that providing alternatives reinforces this need for a visit-ending treatment. “It seems that everybody wants some sort of prescription before they leave. If they don’t get some kind of prescription, whether it’s for Motrin, antibiotics, cough medicine, Tylenol, they don’t feel like they’ve been taken care of.”

Some providers take the time to educate persons about potential hazards of inappropriate use. “I’ll spend a fair amount of time talking about that, placing the importance on that and putting value around that, rather than just saying, "No. You don’t need antibiotics." Which is sort of a negative way of approaching it. I tend to prefer framing it in a positive way.” Others employ rapid tests to help these conversations. For example, one provider stated that RPP can be helpful, **“**If it’s positive for adenovirus or coronavirus, I will tell them, “Look, you have a virus. We know that that does not respond to antibiotics.” And patients are much more amenable once they know what they have**.”**

The capitulation to patient demand is sometimes due to the unyielding nature of some patients. While most providers state that patient demand does not sway their prescribing decisions, some described faltering if they worry about patient satisfaction scores. “You know you’re doing the right thing, but then they make you go the wrong way, because they think they hold the strings in their hands. You know?”

Patient demand was viewed to differ by patient demographics:, “There are occasional people, but it tends to be the kind of patient who is demanding in any way…wanting to dictate whatever their care is…I think it tends to be more the kind of moderately educated, worried well kind of patients that do that as opposed to the bulk of our safety net population.” Education as well as immigration status, were viewed as influencing factors: “…in a lot of places, the only place I’m aware of the most is Latin America, antibiotics are given out… There’s an expectation of receiving treatment…” Additionally some demand is driven by external factors “The major conversation we end up having as pediatricians about prescribing antibiotics is often more for conjunctivitis or pink eye. Some day cares have policies that say until you’re treated with topical antibiotics, you can’t come back. And that gets really tricky because that’s a huge burden on families…

#### 4) Providers do not believe RDTs are implemented with sufficient provider education or evidence for clinical practice

Providers indicated a lack of full awareness of some RDTs and how they should be used. This was particularly prevalent in discussions of RPP and procalcitonin, particularly outside of the ED. One provider stated, “I also know the Procalcitonin, the evidence is not nearly as strong in the outpatient setting, I don’t believe…” and similarly for RPP “…I guess I would be happy to learn more about under what situation in the outpatient world one might use the comprehensive respiratory panel, because I really… I haven’t seen a use for it, but maybe I’m missing something that I should be using it for.” Another provider also illustrated why they thought the clinical value of RPP was lacking “…Like what’s the value of saying somebody’s got an adenovirus or a coronavirus or something like… Who cares? I mean, they have a common upper respiratory viral infection. Like why does it matter which one it is?”

Additionally, some providers described how RDTs were sent for workflow, rather than clinical, reasons. One provider described: “Logistically, to improve workflow, many of the nurses in triage are just making a unilateral decision to send a rapid strep at the time of the admission into the ED even before being roomed or seen by a physician… The vast majority of the time I was walking into the room, seeing those patients either knowing that one had been sent or with a result already for a rapid step.”

## Discussion

We conducted a mixed methods study to examine providers’ knowledge and attitudes towards RDT use, with the goal of identifying barriers and facilitators to appropriate antibiotic prescribing and RDT use. Survey results showed that providers believed they prescribed appropriately and were aware of the clinical antibiotic prescribing guidelines but qualitative interviews indicated that factors such as patient demand influenced prescribing decisions. Providers were generally familiar with the rapid GAS test and the rapid influenza test but only those in the ED and pediatrics showed high familiarity with RPP and procalcitonin. There were inconsistent views on the utility of these tests and during interviews, providers noted that additional evidence was needed to support their use in clinical practice.

Inappropriate antibiotic prescribing for RTIs in the outpatient setting has often been linked to patient demand [6,20]. In our study we found that in all departments except pediatrics, providers felt that patient demand was a significant issue. The reason for the difference is unclear but qualitative interviews suggest that the provider relationship with the patient and ability to educate can determine whether providers give in to demand. Though not significantly higher, providers in pediatrics more often stated that they had enough time to educate their patients. Other studies have found conflicting results on the role of patient demand. Patel et al. showed that high prescribers are more likely to perceive patient demand as an issue.20 In contrast, Gidengil et al. found that providers that did not think patient demand was an issue were more likely to prescribe inappropriately [31]. This suggests that these providers so readily gave antibiotics that their patients did not need to demand them. Taken in conjunction with our findings, these studies highlight that providers play a pivotal role in the effect that patient demand can actually have on antibiotic prescribing.

While the goal of implementing RDTs is to improve antibiotic prescribing, their impact is still unclear. The providers in this study said they trusted the rapid GAS and the rapid influenza tests and believed using them helped make better prescribing decisions. However, there was no consensus on if test results should supersede clinical judgment. Providers explained that if their clinical impression indicated a bacterial infection, they would prescribe an antibiotic regardless of test result. This practice is consistent with findings from our prior EHR analysis where we found that in the presence of a negative rapid GAS test, providers clinically diagnosed 199 cases with streptococcal pharyngitis, 92.5% of which were prescribed an antibiotic [22]. One reason for this practice may be the fact that providers in our survey and other studies state that they are wary of missing a possible bacterial infection [6]. Thus, even in the presence of negative test results and knowledge of prescribing guidelines, providers rely on their clinical assessment of the patient.

Practice location mattered in this study. In terms of RPP, familiarity was highest in the ED and pediatrics, and though our survey found that providers in these departments found RPP useful in prescribing decisions, the qualitative interviews highlighted that views on clinical utility were mixed. One provider noted that a positive viral RPP was useful in explaining to patients why antibiotics were not needed. However, a randomized trial of RPP in ED and inpatient participants, found that there was no difference in the proportion of antibiotics prescribed or mean duration of antibiotic therapy between patients who got RPP and those who did not [32]. Others thought that given that most viruses on the panel did not require treatment, other diagnostic tests such as the rapid influenza test, were more useful. These views were consistent with survey findings, where providers had high familiarity with and trust in rapid influenza results. Further several studies show that the use of rapid influenza testing is associated with increased antiviral prescriptions and in some cases reduced antibiotic prescribing [33–36]. In findings from our previous EHR study, the RPP was primarily used for the influenza result and less so to diagnose other viral etiologies [22]. The uneven use of RPP and views on utility outside of the ED indicates that this particular RDT may only be useful in certain clinical settings.

Of the RDTs examined, overall familiarity with procalcitonin was lowest and providers had limited views on its clinical utility, often citing lack of evidence. In a study in the Netherlands assessing barriers and facilitators for diagnostic tests, though providers were aware of the technical details of the tests, there was still an overwhelming need (70%) for the provision of clinical guidelines to improve use [27]. In a randomized trial in 14 US hospitals, providers were provided with procalcitonin and instructions on the interpretation of results. Analysis found that procalcitonin use was associated with increased prescribing and did not change antibiotic duration or adverse effects [37]. This suggests that the mere provision of testing materials and guideline information is not enough to see appreciable differences in care. Providers need clear evidence that supports using the test will result in improved clinical outcomes.

Our study is not without limitations. Given reports that older providers and those with more clinical experience prescribe more and longer courses of antibiotics than their younger and less experienced colleagues [38,39], it is possible that responses to survey and interview questions may be impacted by age/ clinical experience. Fort-nine percent of our survey sample and 62% of our interviewees were over 45 years old; and 31% of interviewees worked at the institution for over 20 years. Further while we included a diverse sample of providers, we did not include resident physicians. This suggests that responses to the survey and interviews may not accurately represent the views of younger providers with less experience. It is possible that the use of RDTs differs among attending and resident providers and different implementation strategies might be needed to address any issues with uptake. We did not conduct patient interviews and thus we do not know their attitudes towards the acceptability of RDTs. Finally, our sample was relatively small and, as with all qualitative studies, our results may not be generalizable. Despite these limitations, we provide a comprehensive look at RDT use and their role in prescribing at an urban safety net hospital.

## Conclusion

In summary, providers described being knowledgeable about clinical and antibiotic guidelines and are generally aware of RDTs. However, factors such as patient demand, reliance on clinical impressions, and lack of evidence for RDT clinical utility were barriers to appropriate prescribing and RDT use. Our findings highlight the need for implementation strategies that are not only multifaceted, but address the inconsistencies between knowledge and behavior. These may include the inclusion of patient education, tailored approaches for each RDT within different departments and feedback from providers on the integration of RDTs into practice.

## Supporting information

S1 Fig Cabana framework- factors affecting RDT use and antibiotic prescribing. Legend: Codebook based on the Cabana Framework that used for the qualitative analysis of semi-structured interviews.
(DOCX)

S1 Table Survey responses assessing knowledge and attitudes about antibiotic prescribing for respiratory tract infections by department and provider type. (DOCX)

S2 Table Survey responses assessing knowledge and attitudes about rapid diagnostic tests for respiratory tract infections by department and provider type. (DOCX)

S1 Appendix Rapid diagnostic tests outpatient survey. (DOCX)

S2 Appendix Semi-structured interview guide to assess uptake of rapid diagnostic tests for respiratory tract infections and behavioral factors influencing use. (DOCX)
