# Supplementary material for: Provider views on rapid diagnostic tests and antibiotic prescribing for respiratory tract infections: A mixed methods study
Source: PLoS One. 2021 Nov 29;16(11):e0260598. doi: 10.1371/journal.pone.0260598 (PMC8629209; doi:10.1371/journal.pone.0260598)
Supplement: S1 Fig — Legend: Codebook based on the Cabana Framework that used for the qualitative analysis of semi-structured interviews. (DOCX) [file pone.0260598.s001.docx]

**Supplementary Figure 1: Cabana Framework- Factors affecting RDT use and antibiotic prescribing**
