# Supplementary material for: Provider views on rapid diagnostic tests and antibiotic prescribing for respiratory tract infections: A mixed methods study
Source: PLoS One. 2021 Nov 29;16(11):e0260598. doi: 10.1371/journal.pone.0260598 (PMC8629209; doi:10.1371/journal.pone.0260598)
Supplement: S1 Table — (DOCX) [file pone.0260598.s002.docx]

**Supplementary Table 1: Survey responses assessing knowledge and attitudes about antibiotic prescribing for respiratory tract infections by department and provider type**

| **Antibiotic Prescribing (n=85)** | **Disagree** | **Agree** | **Neither Agree nor Disagree** |  | **p value** |
| --- | --- | --- | --- | --- | --- |
| **I am adequately trained to prescribe antibiotics** | | | | |  |
| **Department** |  |  |  |  |  |
| Emergency Department | 1 (4.35) | 22 (95.65) | 0 |  | 0.18 |
| Pediatrics | 0 | 29 (100) | 0 |  |  |
| Other | 0 | 31 (93.94) | 2 (6.06) |  |  |
| **Provider Type** |  |  |  |  |  |
| MD | 0 | 71 (97.26) | 2 (2.74) |  | 0.16 |
| NP | 1 (8.33) | 11 (91.67) | 0 |  |  |
| **I prescribe antibiotics more appropriately than other providers in my department or section. Missing = 1** | | | | |  |
| **Department** | Disagree | Agree | Neither Agree nor Disagree | I don’t know |  |
| Emergency Department | 1 (4.35) | 6 (26.09) | 13 (56.52) | 3 (13.04) | 0.75 |
| Pediatrics | 3 (10.71) | 7 (25) | 12 (42.86) | 6 (21.43) |  |
| Other | 2 (6.06) | 5 (15.15) | 17 (51.52) | 9 (27.27) |  |
| **Provider Type** |  |  |  |  |  |
| MD | 6 (8.33) | 14 (19.44) | 39 (54.17) | 13 (18.06) | 0.11 |
| NP | 0 | 4 (33.33) | 3 (25) | 5 (41.67) |  |
| **It is important to me that my prescribing is consistent with others in my department or section. Missing = 1** | | | | |  |
| **Department** | Disagree | Agree | Neither Agree nor Disagree |  |  |
| Emergency Department | 3 (13.04) | 13 (56.52) | 7 (30.43) |  | 0.3 |
| Pediatrics | 4 (13.79) | 21 (72.41) | 4 (13.79) |  |  |
| Other | 6 (18.75) | 15 (46.88) | 11 (34.38) |  |  |
| **Provider Type** |  |  |  |  |  |
| MD | 10 (13.89) | 44 (61.11) | 18 (25) |  | 0.41 |
| NP | 3 (25) | 5 (41.67) | 4 (33.33) |  |  |
| **I often overprescribe antibiotics.** | | | | |  |
| **Department** | Disagree | Agree | Neither Agree nor Disagree |  |  |
| Emergency Department | 21 (91.3) | 1 (4.35) | 1 (4.35) |  | 0.10 |
| Pediatrics | 24 (87.76) | 0 | 5 (17.24) |  |  |
| Other | 22 (66.67) | 3 (9.09) | 8 (24.24) |  |  |
| **Provider Type** |  |  |  |  |  |
| MD | 57 (78.08) | 3 (4.11) | 13 (17.81) |  | 0.46 |
| NP | 10 (83.33) | 1 (8.33) | 1 (8.33) |  |  |
| **It is a high priority for me not to miss a possible bacterial infection when I select antibiotics to treat my patients.** | | | | |  |
| **Department** | Disagree | Agree | Neither Agree nor Disagree |  |  |
| Emergency Department | 2 (8.7) | 17 (73.91) | 4 (17.39) |  | 0.83 |
| Pediatrics | 2 (6.9) | 22 (75.86) | 5 (17.24) |  |  |
| Other | 2 (6.06) | 28 (84.85) | 3 (9.09) |  |  |
| **Provider Type** |  |  |  |  |  |
| MD | 6 (8.22) | 57 (78.08) | 10 (13.7) |  | 0.74 |
| NP | 0 | 10 (83.33) | 2 (16.67) |  |  |
| **Avoiding adverse effects of antibiotics, such as Clostridium difficile infection, is a major consideration when I prescribe antibiotics.** | | | | |  |
| **Department** | Disagree | Agree | Neither Agree nor Disagree |  |  |
| Emergency Department | 1 (4.35) | 22 (95.65) | 0 |  | 0.16 |
| Pediatrics | 2 (6.9) | 21 (72.41) | 6 (20.69) |  |  |
| Other | 2 (6.06) | 27 (81.82) | 4 (12.12) |  |  |
| **Provider Type** |  |  |  |  |  |
| MD | 4 (5.48) | 59 (80.82) | 10 (13.7) |  | 0.44 |
| NP | 1 (8.33) | 11 (91.67) | 0 |  |  |
| **Patient demand for antibiotics is a significant issue in my practice.** | | | | |  |
| **Department** | Disagree | Agree | Neither Agree nor Disagree |  |  |
| Emergency Department | 8 (34.78) | 12 (52.17) | 3 (13.04) |  | 0.003 |
| Pediatrics | 14 (48.28) | 4 (13.79) | 11 (37.93) |  |  |
| Other | 9 (27.27) | 20 (60.61) | 4 (12.12) |  |  |
| **Provider Type** |  |  |  |  |  |
| MD | 27 (36.99) | 30 (41.1) | 16 (21.92) |  | 0.85 |
| NP | 4 (33.33) | 6 (50) | 2 (16.67) |  |  |
| **Patients are well informed about appropriate antibiotic use.** | | | | |  |
| **Department** | Disagree | Agree | Neither Agree nor Disagree |  |  |
| Emergency Department | 20 (86.96) | 1 (4.35) | 2 (8.7) |  | 0.15 |
| Pediatrics | 16 (55.17) | 3 (10.34) | 10 (34.48) |  |  |
| Other | 23 (69.7) | 4 (12.12) | 6 (18.18) |  |  |
| **Provider Type** |  |  |  |  |  |
| MD | 52 (71.23) | 5 (6.85) | 16 (21.92) |  | 0.17 |
| NP | 7 (58.33) | 3 (25) | 2 (16.67) |  |  |
| **Patients believe antibiotics are necessary for most respiratory tract infections.** | | | |  |  |
| **Department** | Disagree | Agree | Neither Agree nor Disagree |  |  |
| Emergency Department | 3 (13.04) | 18 (78.26) | 2 (8.7) |  | 0.05 |
| Pediatrics | 11 (37.93) | 11 (37.93) | 7 (24.14) |  |  |
| Other | 6 (18.18) | 21 (63.64) | 6 (18.18) |  |  |
| **Provider Type** |  |  |  |  |  |
| MD | 16 (21.92) | 44 (60.27) | 13 (17.81) |  | 0.63 |
| NP | 4 (33.33) | 6 (50) | 2 (16.67) |  |  |
| **I have sufficient time to educate patients when antibiotics are not needed for their infection.** | | | | |  |
| **Department** | Disagree | Agree | Neither Agree nor Disagree |  |  |
| Emergency Department | 11 (47.83) | 7 (30.43) | 5 (21.74) |  | 0.49 |
| Pediatrics | 9 (31.03) | 15 (51.72) | 5 (17.24) |  |  |
| Other | 16 (48.48) | 11 (33.33) | 6 (18.18) |  |  |
| **Provider Type** |  |  |  |  |  |
| MD | 32 (43.84) | 27 (36.99) | 14 (19.18) |  | 0.78 |
| NP | 4 (33.33) | 6 (50) | 2 (16.67) |  |  |
| **I am familiar with clinical guidelines related to antibiotic treatment for acute respiratory tract infections.** | | | | |  |
| **Department** | Disagree | Agree | Neither Agree nor Disagree |  |  |
| Emergency Department | 0 | 22 (95.65) | 1 (4.35) |  | 0.03 |
| Pediatrics | 0 | 29 (100) | 0 |  |  |
| Other | 3 (9.09) | 26 (78.79) | 4 (12.12) |  |  |
| **Provider Type** |  |  |  |  |  |
| MD | 3 (4.11) | 65 (89.04) | 5 (6.85) |  | 1.0 |
| NP | 0 | 12 (100) | 0 |  |  |
| **Patients with acute bronchitis should get antibiotics if their sputum becomes yellow or green.** | | | | |  |
| **Department** | Disagree | Agree | Neither Agree nor Disagree | I don’t know |  |
| Emergency Department | 19 (82.61) | 0 | 4 (17.39) | 0 | 0.99 |
| Pediatrics | 24 (82.76) | 1 (3.45) | 3 (10.34) | 1 (3.45) |  |
| Other | 26 (78.79) | 1 (3.03) | 5 (15.15) | 1 (3.03) |  |
| **Provider Type** |  |  |  |  |  |
| MD | 59 (80.82) | 2 (2.74) | 10 (13.7) | 2 (2.74) | 1.0 |
| NP | 10 (83.33) | 0 | 2 (16.67) | 0 |  |
| **Antibiotics should not be prescribed for clinically stable patients with a comprehensive respiratory panel positive for RSV.** | | | | |  |
| **Department** | Disagree | Agree | Neither Agree nor Disagree | I don’t know |  |
| Emergency Department | 1 (4.35) | 20 (86.96) | 2 (8.7) | 0 | 0.21 |
| Pediatrics | 3 (10.34) | 25 (86.21) | 1 (3.45) | 0 |  |
| Other | 0 | 27 (81.82) | 3 (9.09) | 3 (9.09) |  |
| **Provider Type** |  |  |  |  |  |
| MD | 3 (4.11) | 62 (84.93) | 5 (6.85) | 3 (4.11) | 0.75 |
| NP | 1 (8.33) | 10 (83.33) | 1 (8.33) | 0 |  |
