# Supplementary material for: Provider views on rapid diagnostic tests and antibiotic prescribing for respiratory tract infections: A mixed methods study
Source: PLoS One. 2021 Nov 29;16(11):e0260598. doi: 10.1371/journal.pone.0260598 (PMC8629209; doi:10.1371/journal.pone.0260598)
Supplement: S2 Table — (DOCX) [file pone.0260598.s003.docx]

**Supplementary Table 2: Survey responses assessing knowledge and attitudes about rapid diagnostic tests for respiratory tract infections by department and provider type**

| **I am familiar with rapid diagnostic testing procedures available at Boston Medical Center** | | | | | |
| --- | --- | --- | --- | --- | --- |
| **Comprehensive Respiratory Panel Missing =1** | | | | | |
| **Department** | Disagree | Agree | Neither Agree nor Disagree |  | p value |
| Emergency Department | 0 | 23 (100) | 0 |  | <0.001 |
| Pediatrics | 0 | 29 (100) | 0 |  |  |
| Other | 9 (28.13) | 22 (68.75) | 1 (3.13) |  |  |
| **Provider Type** |  |  |  |  |  |
| MD | 7 (9.72) | 64 (88.89) | 1 (1.39) |  | 0.67 |
| NP | 2 (16.67) | 10 (83.33) | 0 |  |  |
| **Rapid Streptococcal Testing Missing =2** | | | | | |
| **Department** | Disagree | Agree | Neither Agree nor Disagree |  |  |
| Emergency Department | 0 | 23 (100) | 0 |  | 0.45 |
| Pediatrics | 1 (3.57) | 27 (96.43) | 0 |  |  |
| Other | 3 (9.38) | 28 (87.5) | 1 (3.13) |  |  |
| **Provider Type** |  |  |  |  |  |
| MD | 4 (5.63) | 66 (92.96) | 1 (1.41) |  | 1.0 |
| NP | 0 | 12 (100) | 0 |  |  |
| **Rapid Influenza Testing Missing =2** | | | | | |
| **Department** | Disagree | Agree | Neither Agree nor Disagree |  |  |
| Emergency Department | 0 | 23 (100) | 0 |  | 0.07 |
| Pediatrics | 1 (3.45) | 28 (96.55) | 0 |  |  |
| Other | 5 (16.13) | 25 (80.65) | 1 (3.23) |  |  |
| **Provider Type** |  |  |  |  |  |
| MD | 6 (8.45) | 64 (90.14) | 1 (1.41) |  | 0.65 |
| NP | 0 | 12 (100) | 0 |  |  |

| **Procalcitonin Missing = 3** | | | | | |
| --- | --- | --- | --- | --- | --- |
| **Department** | Disagree | Agree | Neither Agree nor Disagree |  |  |
| Emergency Department | 1 (4.35) | 21 (91.3) | 1 (4.35) |  | <0.001 |
| Pediatrics | 7 (25) | 17 (60.71) | 4 (14.29) |  |  |
| Other | 16 (51.61) | 12 (38.71) | 3 (9.68) |  |  |
| **Provider Type** |  |  |  |  |  |
| MD | 20 (28.57) | 44 (62.86) | 6 (8.57) |  | 0.46 |
| NP | 4 (33.33) | 6 (50) | 2 (16.67) |  |  |
| **Rapid diagnostic tests for infectious diseases help me make better antibiotic prescribing decisions.** | | | | | |

| **Comprehensive Respiratory Panel** | | | | | |
| --- | --- | --- | --- | --- | --- |
| **Department** | Disagree | Agree | Neither Agree nor Disagree |  |  |
| Emergency Department | 2 (8.7) | 20 (86.96) | 1 (4.35) |  | 0.02 |
| Pediatrics | 0 | 24 (82.76) | 5 (17.24) |  |  |
| Other | 4 (12.12) | 19 (57.58) | 10 (30.3) |  |  |
| **Provider Type** |  |  |  |  |  |
| MD | 6 (8.22) | 51 (69.86) | 16 (21.92) |  | 0.13 |
| NP | 0 | 12 (100) | 0 |  |  |

| **Rapid Streptococcal Testing** | | | | | |
| --- | --- | --- | --- | --- | --- |
| **Department** | Disagree | Agree | Neither Agree nor Disagree |  |  |
| Emergency Department | 3 (13.04) | 20 (86.96) | 0 |  | 0.15 |
| Pediatrics | 0 | 28 (96.55) | 1 (3.45) |  |  |
| Other | 3 (9.09) | 27 (81.82) | 3 (9.09) |  |  |
| **Provider Type** |  |  |  |  |  |
| MD | 6 (8.22) | 63 (86.3) | 4 (5.48) |  | 0.78 |
| NP | 0 | 12 (100) | 0 |  |  |

| **Rapid Influenza Testing Missing =2** | | | | | |
| --- | --- | --- | --- | --- | --- |
| **Department** | Disagree | Agree | Neither Agree nor Disagree |  |  |
| Emergency Department | 3 (13.64) | 19 (86.36) | 0 |  | 0.10 |
| Pediatrics | 0 | 27 (93.1) | 2 (6.9) |  |  |
| Other | 1 (3.13) | 27 (84.38) | 4 (12.5) |  |  |
| **Provider Type** |  |  |  |  |  |
| MD | 4 (5.63) | 61 (85.92) | 6 (8.45) |  | 0.78 |
| NP | 0 | 12 (100) | 0 |  |  |

| **Procalcitonin** | | | | | |
| --- | --- | --- | --- | --- | --- |
| **Department** | Disagree | Agree | Neither Agree nor Disagree |  |  |
| Emergency Department | 6 (26.09) | 9 (39.13) | 8 (34.78) |  | 0.25 |
| Pediatrics | 2 (6.9) | 11 (37.93) | 16 (55.17) |  |  |
| Other | 5 (15.15) | 9 (27.27) | 19 (57.58) |  |  |
| **Provider Type** |  |  |  |  |  |
| MD | 13 (17.81) | 24 (32.88) | 36 (49.32) |  | 0.33 |
| NP | 0 | 5 (41.67) | 7 (58.33) |  |  |

| **Rapid diagnostic testing results are available quickly enough to help guide my antibiotic prescribing decisions.** | | | | | |
| --- | --- | --- | --- | --- | --- |
| **Comprehensive Respiratory Panel Missing =1** | | | | | |
| **Department** | Disagree | Agree | Neither Agree nor Disagree |  |  |
| Emergency Department | 3 (13.04) | 16 (69.57) | 4 (17.39) |  | 0.02 |
| Pediatrics | 4 (13.79) | 21 (72.41) | 4 (13.79) |  |  |
| Other | 4 (12.5) | 12 (37.5) | 16 (50) |  |  |
| **Provider Type** |  |  |  |  |  |
| MD | 9 (12.5) | 42 (58.33) | 21 (29.17) |  | 0.82 |
| NP | 2 (16.67) | 7 (58.33) | 3 (25) |  |  |

| **Rapid Streptococcal Testing Missing =1** | | | | | |
| --- | --- | --- | --- | --- | --- |
| **Department** | Disagree | Agree | Neither Agree nor Disagree |  |  |
| Emergency Department | 1 (4.35) | 20 (86.96) | 2 (8.7) |  | 0.07 |
| Pediatrics | 0 | 27 (93.1) | 2 (6.9) |  |  |
| Other | 4 (12.5) | 21 (65.63) | 7 (21.88) |  |  |
| **Provider Type** |  |  |  |  |  |
| MD | 3 (4.17) | 59 (81.94) | 10 (13.89) |  | 0.19 |
| NP | 2 (16.67) | 9 (75) | 1 (8.33) |  |  |

| **Rapid Influenza Testing Missing =2** | | | | | |
| --- | --- | --- | --- | --- | --- |
| **Department** | Disagree | Agree | Neither Agree nor Disagree |  |  |
| Emergency Department | 3 (13.64) | 18 (81.82) | 1 (4.55) |  | 0.45 |
| Pediatrics | 3 (10.34) | 23 (79.31) | 3 (10.34) |  |  |
| Other | 4 (12.5) | 21 (65.63) | 7 (21.88) |  |  |
| **Provider Type** |  |  |  |  |  |
| MD | 9 (12.68) | 53 (74.65) | 9 (12.68) |  | 0.88 |
| NP | 1 (8.33) | 9 (75) | 2 (16.67) |  |  |

| **Procalcitonin Missing =4** | | | | | |
| --- | --- | --- | --- | --- | --- |
| **Department** | Disagree | Agree | Neither Agree nor Disagree |  |  |
| Emergency Department | 3 (13.64) | 14 (63.64) | 5 (22.73) |  | 0.01 |
| Pediatrics | 2 (7.14) | 8 (28.57) | 18 (64.29) |  |  |
| Other | 5 (16.13) | 7 (22.58) | 19 (61.29) |  |  |
| **Provider Type** |  |  |  |  |  |
| MD | 8 (11.59) | 27 (39.13) | 34 (49.28) |  | 0.28 |
| NP | 2 (16.67) | 2 (16.67) | 8 (66.67) |  |  |

| **I seldom change antibiotic decisions based on rapid diagnostic testing.** | | | | | |
| --- | --- | --- | --- | --- | --- |
| **Comprehensive Respiratory Panel** | | | | | |
| **Department** | Disagree | Agree | Neither Agree nor Disagree |  |  |
| Emergency Department | 12 (52.17) | 7 (30.43) | 4 (17.39) |  | 0.2 |
| Pediatrics | 9 (31.03) | 12 (41.38) | 8 (27.59) |  |  |
| Other | 8 (24.24) | 12 (36.36) | 13 (39.39) |  |  |
| **Provider Type** |  |  |  |  |  |
| MD | 23 (31.51) | 28 (38.36) | 22 (30.14) |  | 0.46 |
| NP | 6 (50) | 3 (25) | 3 (25) |  |  |
| **Rapid Streptococcal Testing** | | | | | |
| **Department** | Disagree | Agree | Neither Agree nor Disagree |  |  |
| Emergency Department | 8 (34.78) | 11 (47.83) | 4 (17.39) |  | 0.09 |
| Pediatrics | 20 (68.97) | 7 (24.14) | 2 (6.9) |  |  |
| Other | 20 (60.61) | 7 (21.21) | 6 (18.18) |  |  |
| **Provider Type** |  |  |  |  |  |
| MD | 41 (56.16) | 22 (30.14) | 10 (13.7) |  | 1.0 |
| NP | 7 (58.33) | 3 (25) | 2 (16.67) |  |  |
| **Rapid Influenza Testing** | | | | | |
| **Department** | Disagree | Agree | Neither Agree nor Disagree |  |  |
| Emergency Department | 12 (52.17) | 8 (34.78) | 3 (13.04) |  | 0.63 |
| Pediatrics | 15 (51.72) | 10 (34.48) | 4 (13.79) |  |  |
| Other | 15 (45.45) | 9 (27.27) | 9 (27.27) |  |  |
| **Provider Type** |  |  |  |  |  |
| MD | 36 (49.32) | 24 (32.88) | 13 (17.81) |  | 0.84 |
| NP | 6 (50) | 3 (25) | 3 (25) |  |  |
| **Procalcitonin** | | | | | |
| **Department** | Disagree | Agree | Neither Agree nor Disagree |  |  |
| Emergency Department | 4 (17.39) | 11 (47.83) | 8 (34.78) |  | 0.003 |
| Pediatrics | 10 (34.48) | 4 (13.79) | 15 (51.72) |  |  |
| Other | 5 (15.15) | 4 (12.12) | 24 (72.73) |  |  |
| **Provider Type** |  |  |  |  |  |
| MD | 15 (20.55) | 17 (23.29) | 41 (56.16) |  | 0.70 |
| NP | 4 (33.33) | 2 (16.67) | 6 (50) |  |  |
| **I do not trust the results of many of the rapid diagnostic tests.** | | | | | |
| **Comprehensive Respiratory Panel Missing = 1** | | | | | |
| **Department** | Disagree | Agree | Neither Agree nor Disagree |  |  |
| Emergency Department | 22 (95.65) | 0 | 1 (4.35) |  | 0.08 |
| Pediatrics | 25 (86.21) | 2 (6.9) | 2 (6.9) |  |  |
| Other | 23 (71.88) | 1 (3.13) | 8 (25) |  |  |
| **Provider Type** |  |  |  |  |  |
| MD | 59 (81.94) | 3 (4.17) | 10 (13.89) |  | 1.0 |
| NP | 11 (91.67) | 0 | 1 (8.33) |  |  |
| **Rapid Streptococcal Testing Missing =1** | | | | | |
| **Department** | Disagree | Agree | Neither Agree nor Disagree |  |  |
| Emergency Department | 14 (60.87) | 5 (21.74) | 4 (17.39) |  | 0.21 |
| Pediatrics | 24 (82.76) | 3 (10.34) | 2 (6.9) |  |  |
| Other | 23 (71.88) | 2 (6.25) | 7 (21.88) |  |  |
| **Provider Type** |  |  |  |  |  |
| MD | 51 (70.83) | 9 (12.5) | 12 (16.67) |  | 0.88 |
| NP | 10 (83.33) | 1 (8.33) | 1 (8.33) |  |  |
| **Rapid Influenza Testing Missing =1** | | | | | |
| **Department** | Disagree | Agree | Neither Agree nor Disagree |  |  |
| Emergency Department | 21 (91.3) | 0 | 2 (8.7) |  | 0.34 |
| Pediatrics | 24 (82.76) | 1 (3.45) | 4 (13.79) |  |  |
| Other | 22 (68.75) | 3 (9.38) | 7 (21.88) |  |  |
| **Provider Type** |  |  |  |  |  |
| MD | 56 (77.78) | 4 (5.56) | 12 (16.67) |  | 0.83 |
| NP | 11 (91.67) | 0 | 1 (8.33) |  |  |
| **Procalcitonin Missing = 2** | | | | | |
| **Department** | Disagree | Agree | Neither Agree nor Disagree |  |  |
| Emergency Department | 15 (65.22) | 3 (13.04) | 5 (21.74) |  | 0.02 |
| Pediatrics | 20 (68.97) | 1 (3.45) | 8 (27.59) |  |  |
| Other | 14 (45.16) | 0 | 17 (54.84) |  |  |
| **Provider Type** |  |  |  |  |  |
| MD | 41 (57.75) | 4 (5.63) | 26 (36.62) |  | 1.0 |
| NP | 8 (66.67) | 0 | 4 (33.33) |  |  |
| **Rapid diagnostic testing results should never supersede my clinical assessment.** | | | | | |
| **Comprehensive Respiratory Panel Missing =1** | | | | | |
| **Department** | Disagree | Agree | Neither Agree nor Disagree |  |  |
| Emergency Department | 7 (30.43) | 11 (47.83) | 5 (21.74) |  | 0.58 |
| Pediatrics | 13 (44.83) | 9 (31.03) | 7 (24.14) |  |  |
| Other | 9 (28.13) | 13 (40.63) | 10 (31.25) |  |  |
| **Provider Type** |  |  |  |  |  |
| MD | 25 (34.72) | 30 (41.67) | 17 (23.61) |  | 0.39 |
| NP | 4 (33.33) | 3 (25) | 5 (41.67) |  |  |
| **Rapid Streptococcal Testing Missing =1** | | | | | |
| **Department** | Disagree | Agree | Neither Agree nor Disagree |  |  |
| Emergency Department | 6 (26.09) | 13 (56.52) | 4 (17.39) |  | 0.07 |
| Pediatrics | 13 (44.83) | 6 (20.69) | 10 (34.48) |  |  |
| Other | 11 (34.38) | 9 (28.13) | 12 (37.5) |  |  |
| **Provider Type** |  |  |  |  |  |
| MD | 27 (37.5) | 24 (33.33) | 21 (29.17) |  | 0.63 |
| NP | 3 (25) | 4 (33.33) | 5 (41.67) |  |  |
| **Rapid Influenza Testing Missing=2** | | | | | |
| **Department** | Disagree | Agree | Neither Agree nor Disagree |  |  |
| Emergency Department | 7 (30.43) | 12 (52.17) | 4 (17.39) |  | 0.15 |
| Pediatrics | 13 (44.83) | 8 (27.59) | 8 (27.59) |  |  |
| Other | 9 (29.03) | 9 (29.03) | 13 (41.94) |  |  |
| **Provider Type** |  |  |  |  |  |
| MD | 25 (35.21) | 27 (38.03) | 19 (26.76) |  | 0.22 |
| NP | 4 (33.33) | 2 (16.67) | 6 (50) |  |  |
| **Procalcitonin Missing =1** | | | | | |
| **Department** | Disagree | Agree | Neither Agree nor Disagree |  |  |
| Emergency Department | 3 (13.04) | 16 (69.57) | 4 (17.39) |  | 0.002 |
| Pediatrics | 9 (31.03) | 7 (24.14) | 13 (44.83) |  |  |
| Other | 4 (12.5) | 9 (28.13) | 19 (59.38) |  |  |
| **Provider Type** |  |  |  |  |  |
| MD | 14 (19.44) | 30 (41.67) | 28 (38.89) |  | 0.21 |
| NP | 2 (16.67) | 2 (16.67) | 8 (66.67) |  |  |

| **What is the most important factor you consider when deciding whether to order a rapid diagnostic test for your patient?** | | | | | |
| --- | --- | --- | --- | --- | --- |
| **Comprehensive Respiratory Panel** **Missing =5** | | | | | |
| **Department** | **Cost** | **Specificity** | **Sensitivity** | **Time to results** | **Other** |
| Emergency Department | 1 (4.55) | 4 (18.18) | 8 (36.36) | 4 (18.18) | 5 (22.73) |
| Pediatrics | 3 (10.71) | 4 (14.29) | 10 (35.71) | 4 (14.29) | 7 (25) |
| Other | 5 (16.67) | 6 (20) | 7 (23.33) | 7 (23.33) | 5 (16.67) |
| **Provider Type** |  |  |  |  |  |
| MD | 9 (13.24) | 13 (19.12) | 21 (30.88) | 11 (16.18) | 14 (20.59) |
| NP | 0 | 1 (8.33) | 4 (33.33) | 4 (33.33) | 3 (25) |
| **Rapid Streptococcal Testing Missing =4** | | | | | |
| **Department** | **Cost** | **Specificity** | **Sensitivity** | **Time to results** | **Other** |
| Emergency Department |  | 6 (27.27) | 8 (36.36) | 3 (13.64) | 5 (22.73) |
| Pediatrics |  | 7 (25) | 9 (32.14) | 7 (25) | 5 (17.86) |
| Other |  | 5 (16.13) | 13 (41.94) | 8 (25.81) | 5 (16.13) |
| **Provider Type** |  |  |  |  |  |
| MD | 0 | 15 (21.74) | 27 (39.13) | 15 (21.74) | 12 (17.39) |
| NP | 0 | 3 (25) | 3 (25) | 3 (25) | 3 (25) |
| **Rapid Influenza Testing Missing =3** | | | | | |
| **Department** | **Cost** | **Specificity** | **Sensitivity** | **Time to results** | **Other** |
| Emergency Department | 0 | 5 (22.73) | 9 (40.91) | 4 (18.18) | 4 (18.18) |
| Pediatrics | 1 (3.57) | 6 (21.43) | 8 (28.57) | 6 (21.43) | 7 (25) |
| Other | 1 (3.13) | 7 (21.88) | 10 (31.25) | 10 (31.25) | 4 (12.5) |
| **Provider Type** |  |  |  |  |  |
| MD | 2 (2.86) | 17 (24.29) | 23 (32.86) | 16 (22.86) | 12 (17.14) |
| NP | 0 | 1 (8.33) | 4 (33.33) | 4 (33.33) | 3 (25) |
| **Procalcitonin Missing =7** | | | | | |
| **Department** | **Cost** | **Specificity** | **Sensitivity** | **Time to results** | **Other** |
| Emergency Department | 2 (9.09) | 6 (27.27) | 8 (36.36) | 1 (4.55) | 5 (22.73) |
| Pediatrics | 5 (18.52) | 5 (18.52) | 6 (22.22) | 2 (7.41) | 9 (33.33) |
| Other | 2 (6.9) | 6 (20.69) | 6 (20.69) | 7 (24.14) | 8 (27.59) |
| **Provider Type** |  |  |  |  |  |
| MD | 9 (13.43) | 16 (23.88) | 15 (22.39) | 8 (11.94) | 19 (28.36) |
| NP | 0 | 1 (9.09) | 5 (45.45) | 2 (18.18) | 3 (27.27) |
| **What is the least important factor you consider when deciding whether to order a rapid diagnostic test for your patient?** | | | | | |
| **Comprehensive Respiratory Panel** **Missing =6** | | | | | |
| **Department** | **Cost** | **Specificity** | **Sensitivity** | **Time to results** | **Other** |
| Emergency Department | 13 (59.09) | 1 (4.55) | 2 (9.09) | 3 (13.64) | 3 (13.64) |
| Pediatrics | 10 (38.46) | 2 (7.69) | 3 (11.54) | 8 (30.77) | 3 (11.54) |
| Other | 14 (45.16) | 1 (3.23) | 3 (9.68) | 8 (25.81) | 5 (16.13) |
| **Provider Type** |  |  |  |  |  |
| MD | 34 (50) | 4 (5.88) | 5 (7.35) | 15 (22.06) | 10 (14.71) |
| NP | 3 (27.27) | 0 | 3 (27.27) | 4 (36.36) | 1 (9.09) |
| **Rapid Streptococcal Testing Missing =4** | | | | | |
| **Department** | **Cost** | **Specificity** | **Sensitivity** | **Time to results** | **Other** |
| Emergency Department | 14 (63.64) | 2 (9.09) | 0 | 2 (9.09) | 4 (18.18) |
| Pediatrics | 13 (48.15) | 3 (11.11) | 2 (7.41) | 6 (22.22) | 3 (11.11) |
| Other | 17 (53.13) | 2 (6.25) | 2 (6.25) | 7 (21.88) | 4 (12.5) |
| **Provider Type** |  |  |  |  |  |
| MD | 40 (57.14) | 7 (10) | 2 (2.86) | 11 (15.71) | 10 (14.29) |
| NP | 4 (36.36) | 0 | 2 (18.18) | 4 (36.36) | 1 (9.09) |
| **Rapid Influenza Testing Missing =4** | | | | | |
| **Department** | **Cost** | **Specificity** | **Sensitivity** | **Time to results** | **Other** |
| Emergency Department | 13 (59.09) | 2 (9.09) | 0 | 3 (13.64) | 4 (18.18) |
| Pediatrics | 13 (48.15) | 1 (3.7) | 3 (11.11) | 7 (25.93) | 3 (11.11) |
| Other | 16 (50) | 3 (9.38) | 2 (6.25) | 7 (21.88) | 4 (12.5) |
| **Provider Type** |  |  |  |  |  |
| MD | 39 (55.71) | 6 (8.57) | 3 (4.29) | 13 (18.57) | 9 (12.86) |
| NP | 3 (27.27) | 0 | 2 (18.18) | 4 (36.36) | 2 (18.18) |
| **Procalcitonin Missing =9** | | | | | |
| **Department** | **Cost** | **Specificity** | **Sensitivity** | **Time to results** | **Other** |
| Emergency Department | 12 (57.14) | 0 | 0 | 6 (28.57) | 3 (14.29) |
| Pediatrics | 10 (41.67) | 2 (8.33) | 2 (8.33) | 5 (20.83) | 5 (20.83) |
| Other | 14 (45.16) | 3 (9.68) | 3 (9.68) | 5 (16.13) | 6 (19.35) |
| **Provider Type** |  |  |  |  |  |
| MD | 34 (50.75) | 5 (7.46) | 3 (4.48) | 13 (19.4) | 12 (17.91) |
| NP | 2 (22.22) | 0 | 2 (22.22) | 3 (33.33) | 2 (22.22) |
