# Supplementary material for: Provider views on rapid diagnostic tests and antibiotic prescribing for respiratory tract infections: A mixed methods study
Source: PLoS One. 2021 Nov 29;16(11):e0260598. doi: 10.1371/journal.pone.0260598 (PMC8629209; doi:10.1371/journal.pone.0260598)
Supplement: S1 Appendix — (DOCX) [file pone.0260598.s004.docx]

**Rapid Diagnostic Tests Outpatient Survey**

1. I am adequately trained to prescribe antibiotics.

Strongly agree Agree Neither agree or disagree Disagree Strongly disagree

2. I prescribe antibiotics more appropriately than other providers in my department or section.

Strongly agree Agree Neither agree or disagree Disagree Strongly disagree I don't know

3. It is important to me that my prescribing is consistent with others in my department or section.

Strongly agree Agree Neither agree or disagree Disagree Strongly disagree

4. I often overprescribe antibiotics.

Strongly agree Agree Neither agree or disagree Disagree Strongly disagree

5. It is a high priority for me not to miss a possible bacterial infection when I select antibiotics to treat my patients.

Strongly agree Agree Neither agree or disagree Disagree Strongly disagree

6. Avoiding adverse effects of antibiotics, such as Clostridium difficile infection, is a major consideration when I prescribe antibiotics.

Strongly agree Agree Neither agree or disagree Disagree Strongly disagree

7. Patient demand for antibiotics is a significant issue in my practice.

Strongly agree Agree Neither agree or disagree Disagree Strongly disagree

8. Patients are well informed about appropriate antibiotic use.

Strongly agree Agree Neither agree or disagree Disagree Strongly disagree I don't know

9. Patients believe antibiotics are necessary for most respiratory tract infections.

Strongly agree Agree Neither agree or disagree Disagree Strongly disagree I don't know

10. I have sufficient time to educate patients when antibiotics are not needed for their infection.

Strongly agree Agree Neither agree or disagree Disagree Strongly disagree

11. I am familiar with clinical guidelines related to antibiotic treatment for acute respiratory tract infections.

Strongly agree Agree Neither agree or disagree Disagree Strongly disagree

12. Patients with acute bronchitis should get antibiotics if their sputum becomes yellow or green.

Strongly agree Agree Neither agree or disagree Disagree Strongly disagree I don't know

13. Antibiotics should not be prescribed for clinically stable patients with a comprehensive respiratory panel positive for

RSV.

Strongly agree Agree Neither agree or disagree Disagree Strongly disagree I don't know

**Please respond to the following statements for each of the Rapid Diagnostic Tests listed in questions 14-21.**

**14. I am familiar with the rapid diagnostic testing procedures available at Boston Medical**

**Center.**

Strongly agree Agree Neither agree or disagree Disagree Strongly disagree

14a. Comprehensive Respiratory Panel

14b. Rapid Streptococcal Testing

14c. Rapid Influenza Testing

14d. Procalcitonin

**15. Rapid diagnostic tests for infectious diseases help me make better antibiotic prescribing decisions.**

Strongly agree Agree Neither agree or disagree Disagree Strongly disagree

15a. Comprehensive Respiratory Panel

15b. Rapid Streptococcal Testing

15c. Rapid Influenza Testing

15d. Procalcitonin

**16. Rapid diagnostic testing results are available quickly enough to help guide my antibiotic prescribing decisions.**

Strongly agree Agree Neither agree or disagree Disagree Strongly disagree

16a. Comprehensive Respiratory Panel

16b. Rapid Streptococcal Testing

16c. Rapid Influenza Testing

16d. Procalcitonin

**17. I seldom change antibiotic decisions based on rapid diagnostic testing.**

Strongly agree Agree Neither agree or disagree Disagree Strongly disagree

17a. Comprehensive Respiratory Panel

17b. Rapid Streptococcal Testing

17c. Rapid Influenza Testing

17d. Procalcitonin

**18. I do not trust the results of many of the rapid diagnostic tests.**

Strongly agree Agree Neither agree or disagree Disagree Strongly disagree

18a. Comprehensive Respiratory Panel

18b. Rapid Streptococcal Testing

18c. Rapid Influenza Testing

18d. Procalcitonin

**19. Rapid diagnostic testing results should never supersede my clinical assessment.**

Strongly agree Agree Neither agree or disagree Disagree Strongly disagree

19a. Comprehensive Respiratory Panel

19b. Rapid Streptococcal Testing

19c. Rapid Influenza Testing

19d. Procalcitonin

**20. What is the most important factor you consider when deciding whether to order a rapid diagnostic test for your patient?**

Cost Specificity Sensitivity Time to results Other

20a. Comprehensive Respiratory Panel

20b. Rapid Streptococcal Testing

20c. Rapid Influenza Testing

20d. Procalcitonin

If other, please specify:

__________________________________

**21. What is the least important factor you consider when deciding whether to order a rapid diagnostic test for your patient?**

Cost Specificity Sensitivity Time to results Other

21a. Comprehensive Respiratory Panel

21b. Rapid Streptococcal Testing

21c. Rapid Influenza Testing

21d. Procalcitonin

If other, please specify:

__________________________________

**Demographics**

22. Sex

Male Female Transgender Other Prefer not to answer

If other, please specify:

__________________________________

23. What is your race/ethnicity (choose all that apply):

Non-Hispanic White African American/Black Hispanic Asian Other Prefer not to

answer

If other, please specify:

__________________________________

24. Professional degree

MD DO NP PA Other

If other, please specify:

__________________________________

25. Which medical school/professional school did you

attend? __________________________________

26. Where did you complete your residency program?

__________________________________

27. What is your specialty?

__________________________________

28. What is your current department?

__________________________________
