# Supplementary material for: Provider views on rapid diagnostic tests and antibiotic prescribing for respiratory tract infections: A mixed methods study
Source: PLoS One. 2021 Nov 29;16(11):e0260598. doi: 10.1371/journal.pone.0260598 (PMC8629209; doi:10.1371/journal.pone.0260598)
Supplement: S2 Appendix — (DOCX) [file pone.0260598.s005.docx]

**Semi-structured interview guide to assess uptake of rapid diagnostic tests for respiratory tract infections and behavioral factors influencing use**

When assessing a patient how do you decide to prescribe or not prescribe antibiotics for respiratory tract infections?

- 1. What factors go into your decision? Can you walk me though your decision-making process?
  2. Are there certain situations in which you will very often prescribe? Rarely prescribe? Please describe.
  3. How does your prior knowledge of the patient play into this? (are you more likely prescribe to a new patient with an RTI vs. one you know or vice versa or does this matter?)

1. Are there times you find yourself prescribing when you are not really sure the patient needs an antibiotic?
   1. If so, what drives this?
   2. Does the type of RTI play into your decision?
   3. How do patient (or parent, if a child) desires play into your decision? Can you explain? Is it your experience that patients/parents typically desire antibiotics?
   4. Do you think we need to change the patient narrative around antibiotics and if so how can we do so?
2. What has your experience been with the use of rapid diagnostic tests in your outpatient practice?
   1. Have you used certain rapid diagnostic tests over others? If so which ones?
   2. What are the factors that drive your decision to use or not use a rapid diagnostic test?
   3. If you have used them, how, if at all, do they impact or change your prescribing decisions?
3. Although rapid diagnostic tests (procalcitonin, PCR, rapid strep, rapid flu) are available at BMC, in general use of them by providers is low. What are your thoughts about the reason for this lack of use?
   1. We have found that rapid diagnostic test use is particularly low in sinusitis and bronchitis cases, and there is concern about inappropriate prescribing in these illnesses, tell me about your approach to diagnosing and treating a patient with sinusitis or bronchitis.
   2. Are there any barriers to using RDTs that you have personally encountered?
   3. If these barriers were addressed how would this impact your personal use of rapid diagnostic tests?
   4. What do you think can be done to improve uptake of these tests?
   5. In general, what do you think about the utility of these tests and their role in prescribing?
      1. Are they useful in talking to patients about prescribing? How else might they be useful?
4. Is there anything else related to antibiotic prescribing for RTIs and/or use of RDTs related to RTIs that you think is important to mention?
